# Supplementary material for: Multi‐dataset identification of innovative feature genes and molecular mechanisms in keratoconus
Source: J Cell Mol Med. 2024 Sep 19;28(18):e70079. doi: 10.1111/jcmm.70079 (PMC11412914; doi:10.1111/jcmm.70079)
Supplement: Supplementary file 2 — Material S2. [file JCMM-28-e70079-s002.zip › 7.GSEA analysis/2.ATOH7/KEGG_GLYCOSPHINGOLIPID_BIOSYNTHESIS_GANGLIO_SERIES.html]

Details for gene set KEGG\_GLYCOSPHINGOLIPID\_BIOSYNTHESIS\_GANGLIO\_SERIES[GSEA]

|  || Dataset | ATOH7.group.cls#h\_versus\_l |
| Phenotype | group.cls#h\_versus\_l |
| Upregulated in class | l |
| GeneSet | KEGG\_GLYCOSPHINGOLIPID\_BIOSYNTHESIS\_GANGLIO\_SERIES |
| Enrichment Score (ES) | -0.25771302 |
| Normalized Enrichment Score (NES) | -0.7270631 |
| Nominal p-value | 0.74230766 |
| FDR q-value | 1.0 |
| FWER p-Value | 1.0 |
Table: GSEA Results Summary

  

Fig 1: Enrichment plot: KEGG\_GLYCOSPHINGOLIPID\_BIOSYNTHESIS\_GANGLIO\_SERIES      
 Profile of the Running ES Score & Positions of GeneSet Members on the Rank Ordered List

  

| SYMBOL | TITLE | RANK IN GENE LIST | RANK METRIC SCORE | RUNNING ES | CORE ENRICHMENT || 1 | ST3GAL2 | na | 2006 | 0.344 | 0.1998 | No |
| 2 | GLB1 | na | 11405 | 0.060 | -0.0257 | No |
| 3 | ST6GALNAC4 | na | 13855 | 0.032 | -0.0723 | No |
| 4 | HEXB | na | 14910 | 0.022 | -0.0862 | No |
| 5 | HEXA | na | 16012 | 0.013 | -0.1084 | No |
| 6 | ST6GALNAC5 | na | 19977 | -0.022 | -0.2063 | No |
| 7 | ST6GALNAC3 | na | 21762 | -0.040 | -0.2276 | Yes |
| 8 | ST6GALNAC6 | na | 22233 | -0.045 | -0.2071 | Yes |
| 9 | B4GALNT1 | na | 22732 | -0.051 | -0.1830 | Yes |
| 10 | ST3GAL1 | na | 23872 | -0.068 | -0.1651 | Yes |
| 11 | B3GALT4 | na | 24740 | -0.081 | -0.1291 | Yes |
| 12 | ST8SIA5 | na | 25821 | -0.100 | -0.0851 | Yes |
| 13 | ST8SIA1 | na | 26160 | -0.107 | -0.0143 | Yes |
| 14 | SLC33A1 | na | 26248 | -0.109 | 0.0653 | Yes |
| 15 | ST3GAL5 | na | 30776 | -0.239 | 0.1138 | Yes |
Table: GSEA details [plain text format]

  

Fig 2: KEGG\_GLYCOSPHINGOLIPID\_BIOSYNTHESIS\_GANGLIO\_SERIES      
 Blue-Pink O' Gram in the Space of the Analyzed GeneSet

  

Fig 3: KEGG\_GLYCOSPHINGOLIPID\_BIOSYNTHESIS\_GANGLIO\_SERIES: Random ES distribution      
 Gene set null distribution of ES for **KEGG\_GLYCOSPHINGOLIPID\_BIOSYNTHESIS\_GANGLIO\_SERIES**
